# Characteristics of 698 patients with dissociative seizures: A UK multicenter study

**DOI:** 10.1111/epi.16350

**Published:** 2019-10-13

**Authors:** Laura H. Goldstein, Emily J. Robinson, Markus Reuber, Trudie Chalder, Hannah Callaghan, Carole Eastwood, Sabine Landau, Paul McCrone, Nick Medford, John D. C. Mellers, Michele Moore, Iris Mosweu, Joanna Murray, Iain Perdue, Izabela Pilecka, Mark P. Richardson, Alan Carson, Jon Stone, on behalf of the CODES Study Group, Anne‐Mary Abe, Naghme Adab, Niruj Agrawal, Holger Allroggen, Debie Alvares, Thomasin Andrews, Heather Angus‐Leppan, Julia Aram, Richard Armstrong, Antonio Atalaia, Manny Bagary, Masha Bennett, Tamsin Black, Daniel Blackburn, Mayur Bodani, Mark Broadhurst, Alice Brockington, Elisa Bruno, Mary Buckley, Christine Burness, Richard Chalmers, Sam Chong, Muhammad Chowdhury, Fahmida Chowdury, Katia Cikurel, Giovanni Cocco, Hannah Cock, Sarah Cooper, Sarah Cope, Amy Copping, Elana Day, Robert Delamont, Gary Dennis, Christopher Derry, Rebecca Devlin, Jon M Dickson, Beate Diehl, Clare Donnelly, Susan Duncan, Mark Edwards, Shan Ellawella, Cathy Ellis, Jennifer Elvish, Robert Elwes, Sandra Eriemo, Sofia Eriksson, Kerry Evans, Rafey Faruqui, Sarah Feehan, Gerald Finnerty, Lorena Flores, Nick Firth, Robert Fung, Paula Gardiner, Christopher Graham, Zayn Green‐Thompson, Richard Grunewald, Robert Hadden, Khalid Hamandi, Rachel Harding, Sreedharan Harikrishnan, Simon Harrison, Helen Healy, Channa Hewamadduma, Stephen Higgins, Stephen Howell, Heloise Hunt, Abrar Hussain, Motchila Innocente, Graham Jensch, Michael Johnson, Harriet Jordan, Joanna Karlsson, Andrew Kelso, Steven Kemp, Jonathan Knibb, Norman Kock, Michael Koutroumanidis, Stjepana Kovac, Guru Kumar, Andy Laker, Guy Leschziner, Rebecca Liu, Dora Lozsadi, Lea Ludwig, Bridget MacDonald, Lindsey MacGregor, Melissa Maguire, Mark Manford, Davide Martino, Dougall McCorry, Annalisa McGorlick, Kenneth McKeown, Fiona McKevitt, Anna Meadow, Shafqat Memon, Ana Miorelli, Clinton Mitchell, Tejal N Mitchell, Virginia Moffitt, Nicholas Moran, Aimee Morgan‐Boon, John Moriarty, Marco Mula, Nandini Mullatti, Lina Nashef, Daniel O'Hara, Louise Oakley, Suzanne O'Sullivan, Lisa Page, Darshna Patel, Panayiota Petrochilos, Danielle Phoenix, William Pickerell, Thirza Pieters, Norman Poole, Gary Price, David Protheroe, Patrick Pullicino, James Purnell, Jennifer Quirk, Sanjeev Rajakulendran, Julie Read, Basil Ridha, Claire Rockliffe‐Fidler, Carrie Rowbottom, Fergus Rugg‐Gunn, Amrit Sachar, Romi Saha, Gerard Saldanha, Shanika Samarasekera, Violeta Sanchez Sanchez, Alastair Santhouse, Karen Scholes, Abhijeeth Shetty, Paul Shotbolt, Rachel Simkiss, Jasvinder Singh, Jananee Sivagnanasundaram, Sean Slaght, Philip Smith, Dilraj Sokhi, Biba Stanton, Liya Suvorova, Tayyeb Tahir, Ruth Taylor, Lara Teare, Lorenza Tedesco, James Teo, John Thorpe, Laura Toplis, Myrto Tsakopoulou, Ivona Tylova, Tracey Vick, Jane Vinnicombe, Matthew Walker, Cathy Walsh, Gillian Watson, Thomas Webb, Tim Wehner, Killian Welch, Kirstin Weyrich, Margaret Whittaker, Mirdhu Wickremaratchi, Louise Wicks

**Affiliations:** ^1^ Institute of Psychiatry, Psychology, and Neuroscience King's College London London UK; ^2^ School of Population Health and Environmental Sciences King's College London London UK; ^3^ Academic Neurology Unit Royal Hallamshire Hospital University of Sheffield Sheffield UK; ^4^ UK Centre for Clinical Brain Sciences University of Edinburgh Edinburgh UK; ^5^ South London and Maudsley National Health Service Foundation Trust London UK; ^6^ Centre for Social Justice and Global Responsibility School of Law and Social Sciences London South Bank University London UK

**Keywords:** demographics, deprivation, dissociative (nonepileptic) seizures, onset, semiology

## Abstract

**Objective:**

We aimed to characterize the demographics of adults with dissociative (nonepileptic) seizures, placing emphasis on distribution of age at onset, male:female ratio, levels of deprivation, and dissociative seizure semiology.

**Methods:**

We collected demographic and clinical data from 698 adults with dissociative seizures recruited to the screening phase of the CODES (Cognitive Behavioural Therapy vs Standardised Medical Care for Adults With Dissociative Non‐Epileptic Seizures) trial from 27 neurology/specialist epilepsy clinics in the UK. We described the cohort in terms of age, age at onset of dissociative seizures, duration of seizure disorder, level of socioeconomic deprivation, and other social and clinical demographic characteristics and their associations.

**Results:**

In what is, to date, the largest study of adults with dissociative seizures, the overall modal age at dissociative seizure onset was 19 years; median age at onset was 28 years. Although 74% of the sample was female, importantly the male:female ratio varied with age at onset, with 77% of female but only 59% of male participants developing dissociative seizures by the age of 40 years. The frequency of self‐reported previous epilepsy was 27%; nearly half of these epilepsy diagnoses were retrospectively considered erroneous by clinicians. Patients with predominantly hyperkinetic dissociative seizures had a shorter disorder duration prior to diagnosis in this study than patients with hypokinetic seizures (*P *< .001); dissociative seizure type was not associated with gender. Predominantly hyperkinetic seizures were most commonly seen in patients with symptom onset in their late teens. Thirty percent of the sample reported taking antiepileptic drugs; this was more common in men. More than 50% of the sample lived in areas characterized by the highest levels of deprivation, and more than two‐thirds were unemployed.

**Significance:**

Females with dissociative seizures were more common at all ages, whereas the proportion of males increased with age at onset. This disorder was associated with socioeconomic deprivation. Those with hypokinetic dissociative seizures may be at risk for delayed diagnosis and treatment.

Key Points

- In the largest study of dissociative seizures (n = 698), median age at onset was 28 years, but modal age at onset was much younger at 19 years
- Females with dissociative seizures were more common at all ages, but the proportion of males increased with age at onset
- Predominant hyperkinetic semiology was associated with shorter disorder duration prior to diagnosis than hypokinetic semiology
- Dissociative seizure patients were characterized by high levels of socioeconomic deprivation and unemployment in our multicenter cohort

## 1 INTRODUCTION

Difficulties with case ascertainment and sample size have been a barrier to answering questions regarding the demographics of patients with dissociative seizures (also called psychogenic nonepileptic seizures). The recording of typical attacks by video‐electroencephalography (video‐EEG) is considered the diagnostic gold standard, but this test may not be feasible in many cases, for instance because of a low frequency of events, and clinical expertise remains important. Although different levels of diagnostic probability may be clearly defined, there is no objective test that can be relied upon to identify all patients with complete certainty at an early point in the illness trajectory.^1^ As noted elsewhere, the overwhelming majority of studies have been published from single secondary or tertiary care centers,^2^ with local practice and referral bias creating difficulties with generalizability and interpretation.

It has generally been reported that dissociative seizures start with a median age at onset in the mid‐to‐late twenties.^2^, ^3^ However, it has been recognized that older people can also develop the disorder.^4^, ^5^ Previous epidemiological studies suggest that the majority of patients are female, but a different gender ratio has been reported in an older age group.^4^, ^5^

Several studies have suggested that around two‐thirds of dissociative seizures involve predominantly hyperkinetic movements and around one‐third are hypokinetic.^6^, ^7^ A number of studies have found no difference in type of seizure by gender,^3^, ^8^, ^9^, ^10^ age,^4^, ^5^ or culture.^11^ It has been suggested that a history of epilepsy may influence the semiology and phenomenology of dissociative seizures within an individual,^12^ and although there are no data to support or refute this, it has been shown in a between‐groups comparison that dissociative seizure semiology did not differ in groups with or without comorbid or a family history of epilepsy.^13^ Clinical experience suggests that social determinants of ill health such as deprivation and unemployment may also be important in this disorder.^14^ However, surprisingly few data are available on this.

The CODES (Cognitive Behavioural Therapy vs Standardised Medical Care for Adults With Dissociative Non‐Epileptic Seizures) trial was a large multicenter randomized controlled trial (RCT) in the UK evaluating the effect of adding specifically tailored cognitive behavioral therapy (CBT) to standardized medical (including psychiatric) care for dissociative seizures.^15^ It included an initial screening/observational phase, which preceded the main intervention and which presented an opportunity to describe and analyze these variables in a large cohort of patients recruited from 27 centers, overcoming some of the shortcomings of previous epidemiological studies.

We aimed to describe the cohort in terms of age, distribution of age at onset of dissociative seizures (and, in particular, its distribution with respect to gender), duration of seizure disorder, level of socioeconomic deprivation, and other social and clinical demographic characteristics. We also examined associations between variables of interest with respect to gender, predominant dissociative seizure semiology, and age at onset of dissociative seizures.

Specifically, we investigated univariate associations between gender (female vs male), predominant seizure type (hyperkinetic vs hypokinetic), and age at onset (age at first dissociative seizure), as well as each of them with respect to the following binary variables: currently reporting being prescribed antiepileptic drugs (AEDs), having a previous (valid) diagnosis of epilepsy, and having previously sought help for a mental health problem. We also explored the distribution of duration of dissociative seizure disorder (years) with respect to gender and dissociative seizure type.

## 2 MATERIALS AND METHODS

### 2.1 Recruitment

For the first stage of the CODES study, between October 2014 and February 2017, we recruited participants from 27 National Health Service (NHS) secondary/tertiary neurology/epilepsy outpatient clinics. These clinical services were located mainly in London, the South and Southeast of England, Sheffield, Leeds, Birmingham, Edinburgh, and Cardiff. In the UK, the NHS provides free (ie, not insurance‐based and without fee) access to neurological assessment in these centers. Potential participants were identified in these clinics. If a patient met the eligibility criteria (see below) and agreed to be contacted to be given more information about the CODES trial, they were contacted by a research worker who explained the study in greater detail, confirmed eligibility, obtained consent for observational data collection in the initial phase of the study, and collected sociodemographic and clinical information. In this report, we describe data for participants entering this first stage of the study.

Our inclusion criteria for the observation phase of the study were: age ≥ 18 years with dissociative seizures that had occurred within the previous 8 weeks. Dissociative seizures were diagnosed by consultant neurologists and their teams following routine clinical practice.^1^ In cases where only one neurologist had made the diagnosis and video‐EEG telemetry confirmation was unavailable, the diagnosis was then reviewed by epilepsy experts within the CODES team as an additional quality control measure. Other inclusion criteria were: no documented indication of intellectual disability, ability to complete seizure diaries and provide answers to questionnaires, and ability to provide informed consent.

Exclusion criteria were: currently occurring epileptic seizures as well as dissociative seizures (here “currently” was defined as an epileptic seizure experienced in the previous year); meeting criteria for current drug or alcohol dependence based on Diagnostic and Statistical Manual of Mental Disorders, 4th edition^16^ criteria; having insufficiently fluent English skills to complete questionnaires or later on take part in CBT without requiring an interpreter; currently taking part in CBT sessions for another disorder, if this treatment would still be ongoing when the assessment by the psychiatrist occurred; and having formerly undergone a CBT‐based intervention for dissociative seizures at one of the CODES RCT centers.

All participants provided written informed consent. The London‐Camberwell St Giles National Research Ethics Service Committee provided ethical approval for the CODES study (reference number 13/LO/1595).

### 2.2 Measures

We collected the following demographic data from patients: sex, age (and age at first seizure), deprivation status (calculated from online tools using the participant's postcode), relationship status, ethnicity, level of education, employment status, presence of dependents and/or a carer, and whether, if they were of working age, they were in receipt of state‐related financial disability benefits.

The Index of Multiple Deprivation (IMD) uses postcodes to provide an indication of local area deprivation from separate databases for England,^17^ Scotland,^18^ and Wales^19^; we used the databases that were in operation at the time data collection began to maintain consistency, despite newer databases becoming available. The IMD for England and Scotland divides deprivation into specific and quantifiable weighted domains that include employment; income; education, skills, and training; health and disability; crime; living environment; and barriers to housing and services. The IMD for Wales adopts a similar approach but encompasses scores on eight domains: employment, income, education, health, community safety, access to services, physical environment, and housing. Because the databases used also cover somewhat different time periods, IMD scores derived from them cannot simply be combined. The information is collated on the basis of small geographical areas referred to as lower layer super output areas (LSOAs) and allow an individual's level of deprivation to be estimated on the basis of where they live, using their postcode, although deprivation is defined on the basis of neighborhood rather than by the person or their specific household; this is mitigated to some extent by the small size of the LSOAs under consideration. We converted the IMD scores for each dataset into quintiles that were all similarly ordered from least to greatest levels of deprivation. Prior comparisons^20^ show that, although using differing methodology, England and Scotland have been shown to have broadly similar levels of deprivation, whereas Wales has notably higher levels of deprivation, although the percentage of people falling in the quintile indicating greatest deprivation was only slightly higher than for Scotland or England.

Additional variables related to dissociative seizure diagnosis included whether there was a self‐reported previous diagnosis of epilepsy or current prescription of AEDs, and whether participants had sought previous medical treatment for a mental health problem.

Neurologists were asked to record whether the participant's dissociative seizures were predominantly hypokinetic or hyperkinetic. The neurologist was also asked whether the patient had a previous diagnosis of epilepsy, whether the patient still had epilepsy (but had not had an epileptic seizure in the past year), whether the patient had epilepsy when previously diagnosed but now only has dissociative seizures, whether they considered that the patient had been previously misdiagnosed with epilepsy, or whether it was not possible to determine the validity of this earlier diagnosis based upon the available records.

### 2.3 Analysis

Descriptive statistics were reported as medians (interquartile range [IQR]) or frequencies (%), as appropriate. Histograms were used to plot the distribution of a number of continuous variables. A stacked bar chart was used to illustrate aggregated IMD data for England, Scotland, and Wales, where the definition of each quintile varies slightly.

A number of binary variables were also plotted on a connected scatter graph, to demonstrate possible associations with respect to age at onset of dissociative seizures. Deciles of age at onset of dissociative seizures were used instead of standard age‐groupings because of the skewed nature of the variable; this allows for a similar number of patients to be compared between age categories.

To further characterize our sample, we assessed a number of putative associations between some of our variables. Fisher's exact test (two‐sided) was used to test for associations between two categorical (binary) variables, which allowed for the possibility of small cell numbers. Alternatively, to test for associations between binary variables and nonnormally distributed continuous variables, Wilcoxon rank‐sum test was used (also known as Mann‐Whitney).

To be conservative with the potential inflation of a type I error (false positive) from running 14 statistical tests, we used Bonferroni correction to adjust our level of significance (α). This meant that we deemed a statistically significant result as not a chance finding if it had a *P* value < .0036 (0.05/14). We have reported all *P* values prior to correcting for multiple testing.

All analyses were conducted using Stata version 15.0 (StataCorp).

## 3 RESULTS

**Figure 1:**
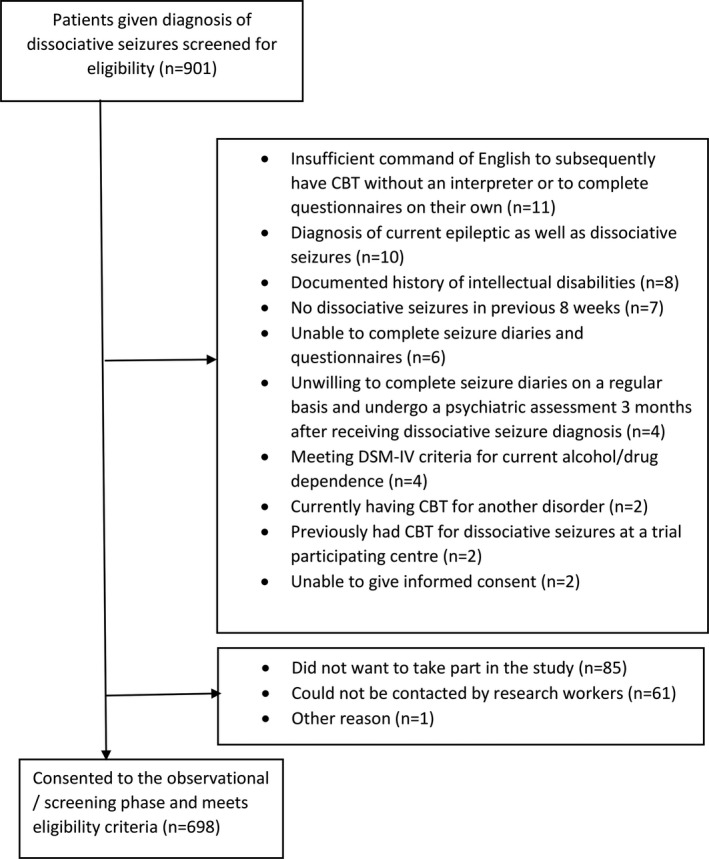
Study flowchart showing the number of patients with dissociative seizures identified in neurology/specialist epilepsy clinics and reasons for ineligibility for the study, leaving 698 recruited to the study. CBT, cognitive behavioral therapy; DSM‐IV, Diagnostic and Statistical Manual of Mental Disorders, 4th edition

We identified 901 patients with dissociative seizures via neurology/specialist epilepsy clinics participating in the CODES trial. From this patient cohort, 845 were eligible to enter the screening phase; a further 147 were excluded (85 did not want to participate, 61 could not be contacted by our research workers, and one was excluded for other reasons). Thus, the total number of patients recruited was 698 (see Figure 1 flowchart).

**Figure 2:**
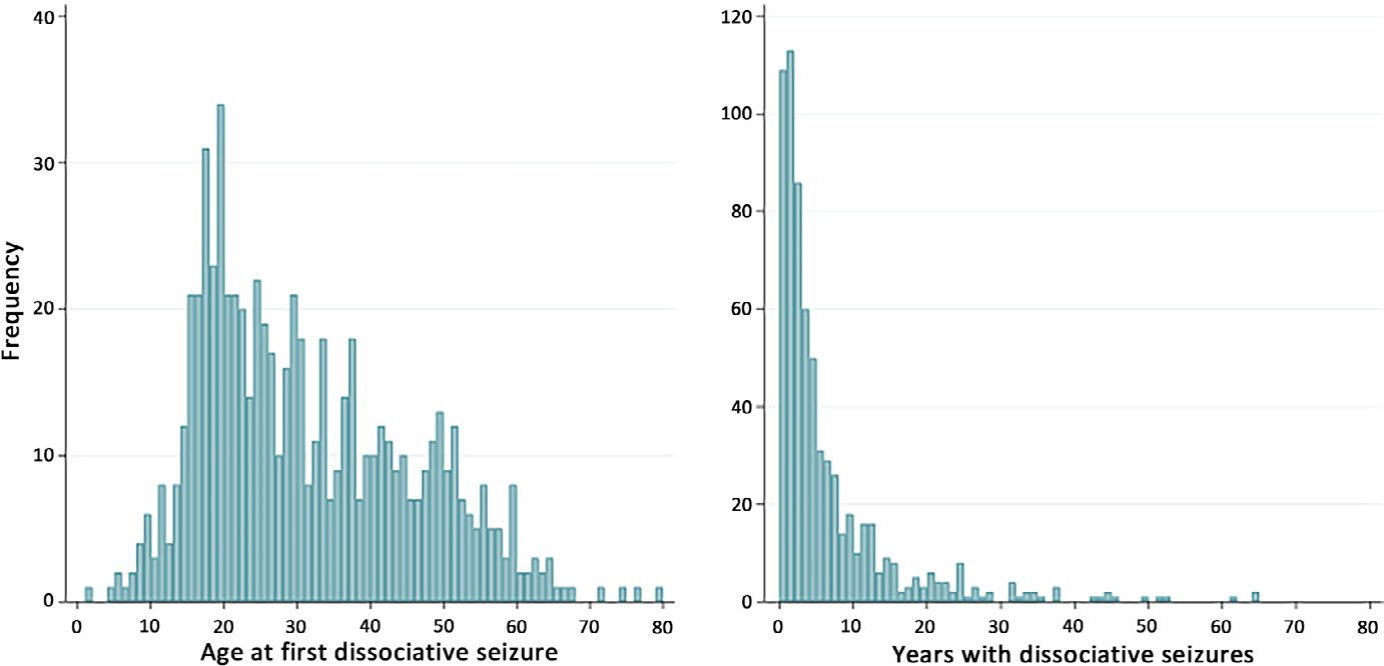
Bar charts showing the frequency distribution of age at onset of dissociative seizures and duration of dissociative seizures in the current sample

The mean age of the participants was 37.1 years, with a median of 34.5 years (IQR = 24‐48 years). The distribution of age at onset is shown in Figure 2. Although median age at onset was 28 years (IQR = 19‐41 years), due to a skewed distribution the modal age at onset was considerably younger at 19 years. There was no evidence of a bimodal distribution.

**Figure 3:**
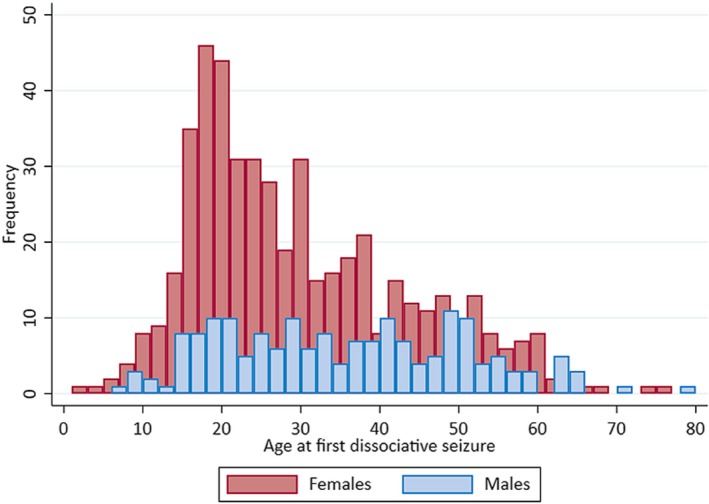
Bar chart showing the frequency distribution of age at first dissociative seizure by gender (females shown in red bars, males in blue bars)

Of the 698 patients, 515 (73.8%) were female. We examined the relationship between age at onset and gender in our cohort. Figure 3 indicates that although nearly three‐quarters of our whole sample were female, the distribution of men and women in our cohort varied considerably according to age at onset. Men had an approximately equal rate of developing dissociative seizures across the age range, whereas women showed a skewed distribution in favor of developing dissociative seizures at a younger age, with 77% of female but only 59% of male participants reporting onset of dissociative seizures before age 40 years.

The median duration of dissociative seizures was 3 years (IQR = 1‐7 years). Figure 2 shows the distribution of duration of the seizure disorder, indicating a long “tail,” with 49/669 (7.3%) patients reporting that their seizure disorder had lasted for >20 years. For ease of comparison with other studies that have not reported median duration of this disorder, the mean duration in this sample was 6.3 years (SD = 9.1; range = 0‐65 years).

**Figure 4:**
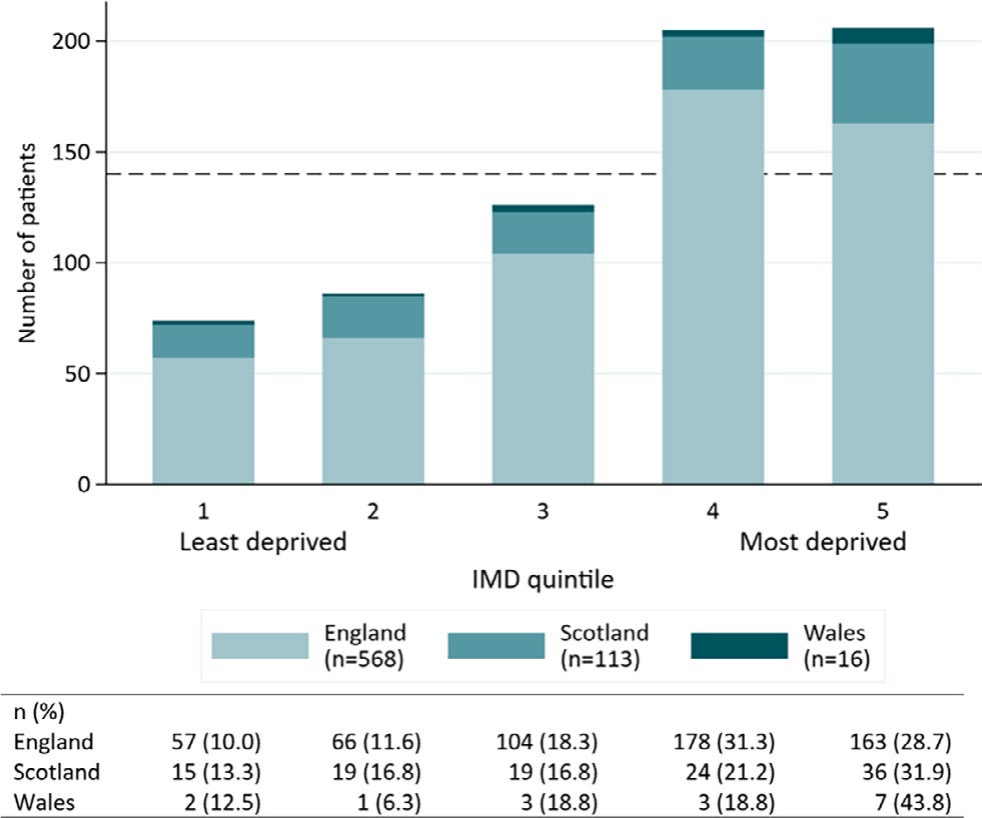
Clustered bar chart illustrating the distribution of quintiles of Index of Multiple Deprivation (IMD) in England, Scotland, and Wales. Quintile 1 represents least deprivation, and quintile 5 represents most deprivation. The horizontal dashed line represents the expected number of people per quintile (140 patients) if the sample is representative of the general population

The distribution of the IMD for participants in England, Scotland, and Wales is shown in Figure 4, demonstrating a clear association between dissociative seizures and socioeconomic deprivation. Although slightly different methods are used to calculate deprivation between the three countries, the resultant classification in England and Scotland is broadly similar. There were only 16 patients for Wales, so we amalgamated the data for England, Scotland, and Wales to provide a summary in Figure 4, showing that >50% of patients with dissociative seizures were in the two most deprived quintiles.

Additional demographics relating to ethnicity, relationship status, living arrangements, highest qualifications obtained, employment status, and disability benefits received are shown in Table 1. The majority of participants were white, married, and living with others. Around one‐third had dependents, and around one‐third reported having a carer who was most often a partner. More than two‐thirds of the sample were unemployed, despite 54.1% having obtained at least secondary or vocational qualifications (and in 30.3% of the cohort, these were further or higher‐level qualifications). Consideration of the subgroup of those aged <65 years revealed that 66% (436/663) were not in education or employment. A high level of dependence on state financial disability benefits (72.9%) was found in those of working age who were not working. Even of those who were working, nearly 20% received supplementary state disability benefits.

**Table 1:** Summaries of sample demographics

	Total number of respondents	Category	n (%)
Ethnicity	697	White	616 (88.4)
		Asian	15 (2.2)
		Black	14 (2.0)
		Mixed/other	52 (7.5)
Relationship status	698	Married/cohabiting	336 (48.1)
		Single	302 (43.3)
		Divorced	29 (4.2)
		Separated	19 (2.7)
		Widowed	12 (1.7)
Who does the participant live with?	698	Living alone	105 (15.0)
		Living with others	593 (85.0)
Does the participant have any dependents?	698	Yes	222 (31.8)
If participant has dependents, they are…^a^	222	Child	211 (95.0)
		Partner	6 (2.7)
		Parent	1 (0.5)
		Other	7 (3.2)
Does participant have a carer?	693	Yes	247 (35.6)
If participant has a carer, they are …^a^	247	Partner	124 (50.2)
		Parent	72 (29.1)
		Child	27 (10.9)
		Friend	21 (8.5)
		Paid	21 (8.5)
		Other	29 (11.7)
Highest educational qualifications obtained	687	No qualifications	107 (15.6)
		Secondary	180 (26.2)
		Vocational	192 (27.9)
		Further	111 (16.2)
		Higher	97 (14.1)
Current employment status^a^	694	Not employed or in education	467 (67.3)
		Employed or in education	227 (32.7)
In receipt of state disability benefits if of working age (<65 y) and not working	446	Yes	325 (72.9)
In receipt of state disability benefits if of working age (<65 y) and working	205	Yes	40 (19.5)

Total can be more than 100%.

a Employed or in education = those who were employed full‐ or part‐time time (and working) or who were students or who were self‐employed; not employed or in education = those who were unemployed, those who were employed full‐time or part‐time but off sick, students whose studies were interrupted due to illness, those who were retired due to age or ill health, and those who were a housewife/househusband.

Four hundred seventy‐one (68%) patients had seizures that were recorded by their neurologist as predominantly hyperkinetic, and 221 (32%) were recorded as having mainly hypokinetic seizures. One hundred eighty‐eight patients (27%) self‐reported a previous diagnosis of epilepsy. Of these 188 patients, neurologists commented on 179 of their epilepsy diagnoses: only 15 were judged by the neurologist to still have epilepsy but not to have had an epileptic seizure in the past year, 20 were thought to have previously had an accurate diagnosis of epilepsy but to have only dissociative seizures now, 80 were thought to have previously been misdiagnosed with epilepsy, and in 64 it was not possible to determine the validity of the diagnosis of epilepsy. Although the figure for dual diagnosis was relatively high at 30% (35/115), only 13% were considered to have ongoing (but not currently active) epilepsy. Two hundred eleven (30%) of the total sample of 698 participants self‐reported being on AEDs.

Four hundred fifty‐three people (65%) said they had previously sought help for a mental health problem.

Table 2 shows the relationships of gender and predominant type of seizure with age at onset of dissociative seizures, duration of disorder, reporting taking AEDs, existence of a previous diagnosis of epilepsy, and reporting having previously sought help for mental health problems. Participants for whom the diagnoses of epilepsy were thought to be erroneous or could not be substantiated were excluded from this group classified as having epilepsy. Men had an older median age at onset of dissociative seizures (34.5 vs 26; *P* < .001) and were more likely to report taking AEDs than women (37.4% vs 28.6%; *P* = .032), with the difference in age at onset between genders surviving Bonferroni correction. In addition, those with predominantly hyperkinetic seizures had a shorter median duration of their dissociative seizure disorder compared to predominantly hypokinetic seizures (2 years vs 4 years; *P* < .001). There was no association between age at onset of dissociative seizures and currently being prescribed AEDs (*P* = .107), having a previous (valid) diagnosis of epilepsy (*P* = .358), or previously having sought help for a mental health problem (*P* = .651).

**Table 2:** Associations with gender, predominant seizure type, and age at onset of dissociative seizures

	Female, n = 515	Male, n = 182	*P*	Predominantly hyperkinetic, n = 471	Predominantly hypokinetic, n = 221	*P*	Age at onset, n = 669	*P*
Age at onset	n = 493	n = 176	*P* < .001^a^	n = 448	n = 216	*P* = .101	—	—
Median (IQR)	26 (19‐38)	34.5 (22‐48)		30 (21‐43)	26 (19‐40)			
Years with dissociative seizures	n = 493	n = 176	*P* = .378	n = 448	n = 216	*P* < .001^a^	—	—
Median (IQR)	3 (1‐7)	3 (1‐8)		2 (1‐7)	4 (2‐10)			
Predominant seizure type, n (%)	n = 511	n = 181	*P* = .354	—	—	—	—	—
Hypokinetic	158 (30.9)	63 (34.8)						
Hyperkinetic	353 (69.1)	118 (65.2)						
Currently prescribed AEDs, n (%)	n = 514	n = 182	*P* = .032^b^	n = 469	n = 221	*P* = .427	n = 668	*P* = .107
Yes	147 (28.6)	68 (37.4)		148 (31.6)	63 (28.5)		29, IQR = 20‐44	
No	367 (71.4)	114 (62.6)		321 (68.4)	158 (71.5)		28, IQR = 19‐40	
Previous valid diagnosis of epilepsy, n (%)	n = 473	n = 152	*P* = .686	n = 419	n = 201	*P* = .266	n = 599	*P* = .358
Yes	28 (5.9)	7 (4.6)		27 (6.4)	8 (4.0)		31, IQR = 20‐48	
No	445 (94.1)	145 (95.4)		392 (93.6)	193 (96.0)		28, IQR = 19‐41.5	
Previously sought help for a mental health problem, n (%)	n = 515	n = 182	*P* = .148	n = 470	n = 221	*P* = .797	n = 669	*P* = .651
Yes	343 (66.6)	110 (60.4)		308 (65.5)	142 (64.3)		28, IQR = 19‐40	
No	172 (33.4)	72 (39.6)		162 (34.5)	79 (35.7)		27, IQR = 18.5‐42	

Abbreviations: AEDs, antiepileptic drugs; IQR, interquartile range.

^a^Remains significant after Bonferroni correction (α < .0036).

^b^Statistically significant at α < .05 level.

**Figure 5:**
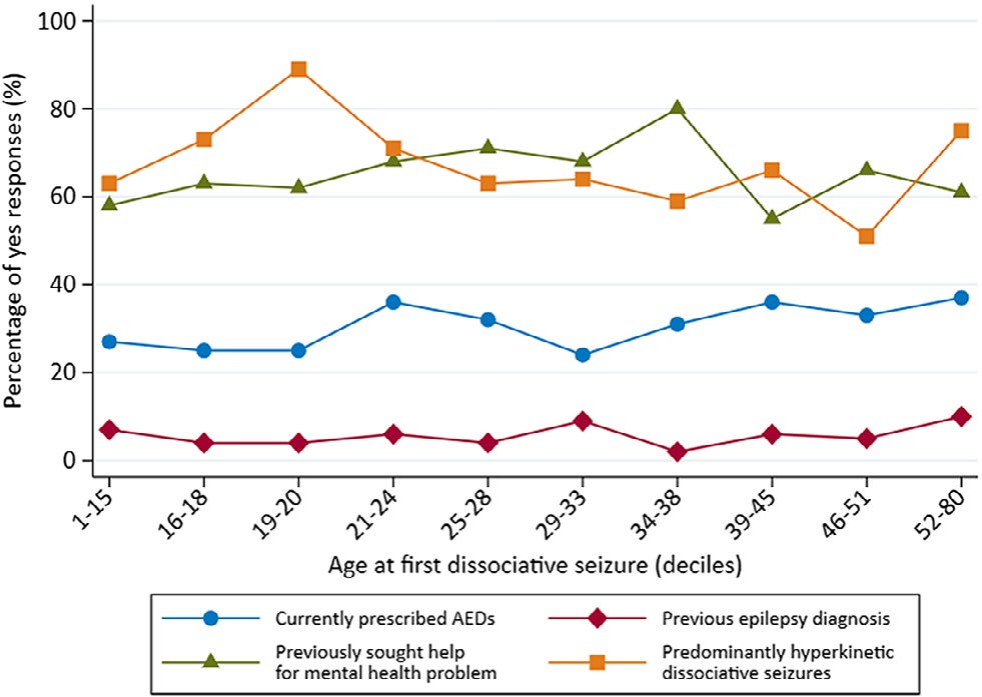
Line graphs showing percentages of the sample reporting current antiepileptic drug (AED) use, having a previous (valid) diagnosis of epilepsy, self‐reporting previous help‐seeking for a mental health problem, and having clinician‐rated predominantly hyperkinetic dissociative seizures, in relation to age at onset of dissociative seizures

Figure 5 shows age at onset (in deciles) plotted against percentage of participants reporting current AED prescription, predominant hyperkinetic seizure type, previous (valid) epilepsy diagnosis, and having sought help for mental health problems. We observed a peak of predominantly hyperkinetic dissociative seizure events in the age 19‐20 decile onset.

## 4 DISCUSSION

Examination of the demographic data within this large cohort of patients highlighted new and important insights into the demographics and characteristics of people with dissociative seizures. Along with other researchers,^2^ we found a median age at onset of 28 years, but the commonest, modal, age at onset was nearly a decade younger, at 19 years. Dissociative seizures were much more common in females, but the median age at onset differs considerably in men and women, because there is a large age‐related incidence peak in women younger than 40 years, whereas the age‐related incidence in men varies little across the adult lifespan. Our current data did not allow us to identify factors relating to possible abuse histories that may have differed systematically between our male and female participants; our data showed a nonsignificant trend (after Bonferroni correction) for men with dissociative seizures to be more likely to be prescribed AEDs, raising the possibility of medical factors being more pertinent for men than women in the sample. However, given that the diagnoses of additional epilepsy were thought to be incorrect in nearly one‐half of all patients self‐reporting this comorbidity, and were not formally confirmed in the majority of the remainder (for instance by video‐EEG or consensus review of all available records), it may also be that gender differences in AED prescribing were a reflection of different levels of diagnosticians’ certainty in men and women or of a degree of gender bias in neurologists’ treatment choices.

Our cohort of patients with dissociative seizures was characterized by high levels of socioeconomic deprivation. Allowing for the differences in the three IMD databases used, more than one‐half of the sample fell in the two quintiles representing the highest levels of deprivation. In addition, the group was characterized by high levels of unemployment, and for those of working age, the majority were dependent on state financial benefits. UK data (albeit based on adults aged 16‐64 years of age) suggest that, for example in 2015 and 2016 (during the lifetime of the study), between 21% and 22% were classed as economically inactive,^21^ whereas for our sample (aged 18‐84 years), 67% could be classed as economically inactive. Here we define “economically inactive” as those who were unemployed, employed full‐time or part‐time but off sick, students whose studies were interrupted due to illness, a housewife/househusband, or retired due to age or ill‐health. Being more selective, we also found that of those aged <65 years, 66% were neither in employment nor in education. Furthermore, whereas national figures suggest that 19% of people of working age report a disability, in our sample 73% of those of working age reported receiving state disability benefits, thereby suggesting a much higher level of reported disability in study participants of working age than the national average.^22^ The high levels of deprivation and benefits use associated with the dissociative seizures diagnosis in this population clearly demonstrate the social and societal dimension of this disorder, which may have effects on access to diagnostic and treatment services and diagnostic delay and may be relevant in terms of etiology, suitable interventions, and outcome. The high levels of deprivation replicated and extended the findings of Duncan et al,^14^ who reported on a large series of patients in Glasgow, UK. Nonetheless, it should be emphasized that the demographic spread of presentations was wide, and that having dissociative seizures represents a disorder that can potentially affect anyone, irrespective of age and gender.

The distribution of duration of dissociative seizures in this cohort was highly skewed, with 7.3% reporting dissociative seizures that had been present for >20 years by the time they were recruited to our study. Most other studies have reported mean (rather than median) duration of this disorder, and the mean duration of dissociative seizures in our sample is consistent with summaries of other reports.^2^ In contrast to an earlier study indicating no relationship between dissociative seizure semiology and diagnostic delay,^23^ in this study, dissociative seizure duration was associated with predominant seizure semiology. Thus, those with predominantly hyperkinetic seizures had a seizure disorder of more recent onset than those with predominantly hypokinetic dissociative seizures. This may be because patients with hyperkinetic seizures are more likely to present to emergency services and less likely to be initially managed as having simple faints. Our findings also indicated a potential peak of predominantly hyperkinetic dissociative seizure events in the age 19‐20 years onset decile (Figure 5). This was consistent with the modal age at onset of dissociative seizures in the cohort as a whole (Figure 2) and the younger median age at onset of patients generally with predominantly hyperkinetic versus hypokinetic events (26 years vs 30 years; Table 2).

We did not find gender differences in those with different seizure types, consistent with other findings.^3^, ^8^, ^9^, ^10^ Once we excluded patients whose prior epilepsy diagnoses were likely to have been incorrect or could not be verified, we failed to find a relationship between dissociative seizure type and epilepsy history. Although this negative finding could be due to low statistical power, we did not have details of the classification of patients’ epilepsy. We therefore cannot rule out that a history of seizures could influence dissociative seizure semiology.^12^

We acknowledge a number of limitations in our study, which was not a population study and cannot claim to have captured a truly consecutive patient cohort. Our group of 698 was recruited in the context of a pragmatic RCT with specific inclusion and exclusion criteria identifying patients in secondary/tertiary care neurology/specialist epilepsy clinics that also led to the inclusion of patients who had not received their diagnoses using video‐EEG telemetry. Although video‐EEG telemetry is the gold standard for diagnosis, it is not always widely or rapidly available. A proportion of patients do not have their dissociative seizures while on telemetry in any case. When no video‐EEG confirmation was available, patients were only included if the diagnosis was additionally confirmed by another consultant who had seen the patient or by a consultant with access to relevant clinical data. Our participants were those willing to be involved in a potentially lengthy study that might have led them to receive psychological therapy, and all received a leaflet about dissociative seizures from their diagnosing neurologist^15^; we cannot estimate how our current sample differed from those declining to take part, but acknowledge some potential self‐selection bias. We excluded people with an epileptic seizure in the previous year, but even so, 27% of our included patients self‐reported having had a previous diagnosis of epilepsy. Neurological assessment questioned the validity of many these diagnoses, but nonetheless we included some patients (13%) in whom the diagnosis was believed to be accurate. These data strongly support previous claims of the likelihood of misdiagnosis of prior epilepsy in patients with dissociative seizures.^2^ Our sample also excluded people with insufficient proficiency in English to participate in the study without the aid of an interpreter, and the overwhelming majority in the sample was white. The exclusion of patients with substance misuse problems and intellectual disability may have introduced further bias. In addition, many of our recruiting clinical services were in urban areas, potentially influencing the level of deprivation of patients. The cross‐sectional nature of this investigation means that we cannot determine whether the associations of dissociative seizures with other observed features (eg, socioeconomic status) were causally related and, if so, in which direction.

Despite these limitations, we nonetheless have data on almost 700 patients who have had thorough assessment from 27 centers across the UK. This study has highlighted novel findings in the basic epidemiology of dissociative seizures. Although it remains a disorder predominantly affecting women, with onset across the age range,^2^ the likelihood of developing this disorder is not equal across ages in men and women. The distribution of age at onset is highly skewed, and whereas the median age is 28 years (not dissimilar from the mean age at onset reported across studies from different countries^2^), the single commonest age at onset is 19 years, apparently accompanied by a greater likelihood of having predominantly hyperkinetic rather than hypokinetic seizures at this age. It is a disorder associated with socioeconomic deprivation, and likely misdiagnosis with epilepsy is common.

## 5 IMPLICATIONS

Understanding the characteristics of a large sample of patients with dissociative seizures has important implications for interpretation of suggested etiological factors and treatment, especially those factors that are confounded with the gender, age, and socioeconomic groups from which dissociative seizure patients are most likely to emerge.

## 7 CONFLICT OF INTEREST

A.C. reports being a paid editor of the *Journal of Neurology, Neurosurgery, and Psychiatry*, and is the director of a research program on functional neurological disorders; he gives independent testimony in court on a range of neuropsychiatric topics (50% pursuer, 50% defender). M.P.R. reports funding from Xenon Pharma. J.S. reports independent expert testimony work for personal injury and medical negligence claims, and royalties from UpToDate for articles on functional neurological disorder, and runs a free nonprofit self‐help website, http://www.neurosymptoms.org. None of the other authors has any conflict of interest to disclose. We confirm that we have read the Journal's position on issues involved in ethical publication and affirm that this report is consistent with those guidelines.

## APPENDIX 1

### 1.1 CODES Study Group

Anne‐Mary Abe, Naghme Adab, Niruj Agrawal, Holger Allroggen, Debie Alvares, Thomasin Andrews, Heather Angus‐Leppan, Julia Aram, Richard Armstrong, Antonio Atalaia, Manny Bagary, Masha Bennett, Tamsin Black, Daniel Blackburn, Mayur Bodani, Mark Broadhurst, Alice Brockington, Elisa Bruno, Mary Buckley, Christine Burness, Richard Chalmers, Sam Chong, Muhammad Chowdhury, Fahmida Chowdury, Katia Cikurel, Giovanni Cocco, Hannah Cock, Sarah Cooper, Sarah Cope, Amy Copping, Elana Day, Robert Delamont, Gary Dennis, Christopher Derry, Rebecca Devlin, Jon M. Dickson, Beate Diehl, Clare Donnelly, Susan Duncan, Mark Edwards, Shan Ellawella, Cathy Ellis, Jennifer Elvish, Robert Elwes, Sandra Eriemo, Sofia Eriksson, Kerry Evans, Rafey Faruqui, Sarah Feehan, Gerald Finnerty, Lorena Flores, Nick Firth, Robert Fung, Paula Gardiner, Christopher Graham, Zayn Green‐Thompson, Richard Grunewald, Robert Hadden, Khalid Hamandi, Rachel Harding, Sreedharan Harikrishnan, Simon Harrison, Helen Healy, Channa Hewamadduma, Stephen Higgins, Stephen Howell, Heloise Hunt, Abrar Hussain, Motchila Innocente, Graham Jensch, Michael Johnson, Harriet Jordan, Joanna Karlsson, Andrew Kelso, Steven Kemp, Jonathan Knibb, Norman Kock, Michael Koutroumanidis, Stjepana Kovac, Guru Kumar, Andy Laker, Guy Leschziner, Rebecca Liu, Dora Lozsadi, Lea Ludwig, Bridget MacDonald, Lindsey MacGregor, Melissa Maguire, Mark Manford, Davide Martino, Dougall McCorry, Annalisa McGorlick, Kenneth McKeown, Fiona McKevitt, Anna Meadow, Shafqat Memon, Ana Miorelli, Clinton Mitchell, Tejal N. Mitchell, Virginia Moffitt, Nicholas Moran, Aimee Morgan‐Boon, John Moriarty, Marco Mula, Nandini Mullatti, Lina Nashef, Daniel O'Hara, Louise Oakley, Suzanne O'Sullivan, Lisa Page, Darshna Patel, Panayiota Petrochilos, Danielle Phoenix, William Pickerell, Thirza Pieters, Norman Poole, Gary Price, David Protheroe, Patrick Pullicino, James Purnell, Jennifer Quirk, Sanjeev Rajakulendran, Julie Read, Basil Ridha, Claire Rockliffe‐Fidler, Carrie Rowbottom, Fergus Rugg‐Gunn, Amrit Sachar, Romi Saha, Gerard Saldanha, Shanika Samarasekera, Violeta Sanchez Sanchez, Alastair Santhouse, Karen Scholes, Abhijeeth Shetty, Paul Shotbolt, Rachel Simkiss, Jasvinder Singh, Jananee Sivagnanasundaram, Sean Slaght, Philip Smith, Dilraj Sokhi, Biba Stanton, Liya Suvorova, Tayyeb Tahir, Ruth Taylor, Lara Teare, Lorenza Tedesco, James Teo, John Thorpe, Laura Toplis, Myrto Tsakopoulou, Ivona Tylova, Tracey Vick, Jane Vinnicombe, Matthew Walker, Cathy Walsh, Gillian Watson, Thomas Webb, Tim Wehner, Killian Welch, Kirstin Weyrich, Margaret Whittaker, Mirdhu Wickremaratchi, Louise Wicks, Mahinda Yogarajah.
